# Characterization of the soil resistome and mobilome in Namib Desert soils

**DOI:** 10.1007/s10123-023-00454-x

**Published:** 2023-11-16

**Authors:** Yashini Naidoo, Rian E. Pierneef, Don A. Cowan, Angel Valverde

**Affiliations:** 1 Centre for Microbial Ecology and Genomics, Department of Biochemistry, Genetics and Microbiology, University of Pretoria, Lynnwood Road, Pretoria, 0002 South Africa; 2 Biotechnology Platform, Agricultural Research Council, Soutpan Road, Onderstepoort Campus, Pretoria, 0110 South Africa; 3 IRNASA-CSIC, Cordel de Merinas, 37008 Salamanca, Spain

**Keywords:** Resistome, Namib Desert, Mobile genetic elements, Antibiotic resistance

## Abstract

The study of the soil resistome is important in understanding the evolution of antibiotic resistance and its dissemination between the clinic and the environment. However, very little is known about the soil resistome, especially of those from deserts. Here, we characterize the bacterial communities, using targeted sequencing of the 16S rRNA genes, and both the resistome and the mobilome in Namib Desert soils, using shotgun metagenomics. We detected a variety of antibiotic resistance genes (ARGs) that conferred resistance to antibiotics such as elfamycin, rifampicin, and fluoroquinolones, metal/biocide resistance genes (MRGs/BRGs) conferring resistance to metals such as arsenic and copper, and mobile genetic elements (MGEs) such as the ColE1-like plasmid. The presence of metal/biocide resistance genes in close proximity to ARGs indicated a potential for co-selection of resistance to antibiotics and metals/biocides. The co-existence of MGEs and horizontally acquired ARGs most likely contributed to a decoupling between bacterial community composition and ARG profiles. Overall, this study indicates that soil bacterial communities in Namib Desert soils host a diversity of resistance elements and that horizontal gene transfer, rather than host phylogeny, plays an essential role in their dynamics.

**Supplementary Information:**

The online version contains supplementary material available at 10.1007/s10123-023-00454-x.

## Introduction

The misuse and overuse of antibiotics in the human, animal, and agricultural sectors are an important selective force in the evolution and the dissemination of antibiotic resistance (Bhullar et al., 2012; von Wintersdorff et al., 2016), which is one of the major global challenges of the twenty-first century as it poses serious risks to human health. The One Health concept is a holistic and interdisciplinary approach based on the idea that human and animal health are linked to the health of the ecosystems that they are a part of (Hernando-Amado et al., 2019). The importance of a One Health approach to antibiotic resistance (AR) is clear especially with respect to the dissemination of antibiotic resistance genes that have crossed habitat boundaries (Naidoo et al., 2020).

Soils are perhaps the most significant reservoir of ARGs as many soil microbes produce natural antibiotics to outcompete other microbes (Nesme and Simonet, 2015; Zhu et al., 2019). Indeed, most clinically relevant antibiotics such us streptomycin, tetracycline, and vancomycin originate from soil-dwelling actinomycetes (D’Costa et al., 2006; Cytryn, 2013). Microorganisms have developed a variety of resistance mechanisms (encoded by resistance genes) to prevent them from succumbing to these toxic metabolites (Thaker et al., 2013; Jiang et al., 2017). Many bacteria are naturally resistant to a broad spectrum of antibiotics (Demaneche et al., 2008; Allen et al., 2009), which may reflect their ability to produce more than one antibiotic, or be a by-product of their evolution in proximity to antibiotic-producing neighboring bacteria (Perry and Wright, 2013). Consequently, it is now clear that the soil environment harbors a plethora of both discovered and undiscovered resistance genes, which together constitute the soil *resistome* (Dantas and Sommer, 2012; Gillings et al., 2017).

Most types of soils contain heavy metals, some of which (at low concentrations) contribute to the metabolism of microorganisms (Knapp et al., 2017; Chen et al., 2019). Interestingly, metals can act as co-selective forces contributing to the increase in antibiotic resistance (Knapp et al., 2017). The relative importance of co-selection of resistance to both antibiotics and metals/biocides is likely to be different in different environments. For instance, some desert soils are known to have low anthropogenic antibiotic input but relatively high levels of metals over long periods of time (Knapp et al., 2011). Consequently in deserts, even in the absence of antibiotics, metals may provide a stronger and more persistent selective pressure for the environmental selection of antibiotic resistance (Zhao et al., 2018).

Resistance genes can move between soil microbial taxa via mobile genetic elements, collectively known as the *mobilome* (Carr et al., 2020). The mobilome facilitates the acquisition of traits between bacteria through several mechanisms via horizontal gene transfer (HGT), that is, via transformation (involving free DNA), transduction (involving bacteriophages), or conjugation (involving plasmids and integrative conjugative elements) (Peterson and Kaur, 2018). Soil bacteria undergo higher rates of gene transfer in “hotspots” (i.e., areas of higher nutritional content) such as the rhizosphere and manure-treated soil (Perry and Wright, 2013). However, the prevalence of horizontal gene transfer in native soil microbial communities and the effects it may have on soil processes are largely unknown and require further investigation (Fierer, 2017).

It has been hypothesized that many ARGs found in clinical isolates have originated from soil (Forsberg et al., 2012; Walsh and Duffy, 2013; McCann et al., 2019). Therefore, investigating the soil resistome could enable the detection of clinically relevant antibiotic resistance mechanisms (Forsberg et al., 2012; Walsh and Duffy, 2013; McCann et al., 2019). Furthermore, tracking antibiotic resistance genes in less impacted (i.e., deserts) or unimpacted (pristine) soils is important because this allows for the detection of background or intrinsic levels of antibiotic resistance in soil which may aid in estimating potential human health risks (Scott et al., 2020). Additionally, it might help to elucidate the extent of contamination of ARGs due to anthropogenic activity.

In this study, using targeted sequencing of the 16S rRNA genes, we characterized the bacterial communities and, using shotgun metagenomics, the soil resistome and mobilome in surface soils from the Namib Desert. The aims of this study were to investigate (1) the diversity and composition of ARGs and metal resistance genes/biocide resistance genes (MRGs/BRGs), (2) whether or not horizontal gene transfer (HGT) affected the distribution of the resistome, (3) possible co-selection of resistance with metals/biocides and antibiotics, and (4) a possible link between microbial community composition and the resistome.

## Experimental procedures

### Sampling, soil chemistry, and climate data

Eighteen surface soils (0 to 5 cm deep) were collected across a transect in the Namib Desert. Previously, anthropogenic influences across the desert have been minimal and were mostly limited to scientific expeditions. However, the nature-based tourism has increased drastically in the last decade, possibly increasing the anthropogenic pressure in the Namib Desert (Naidoo et al., 2020). The 18 sampling sites were spaced 5–10 km apart, and at each site, four aliquots of 50g of soil were taken at 100 m spacing and combined in a composite sample. Soils were collected using sterile methods and stored in sterile 50 ml polypropylene Falcon tubes (Grenier, Bio-One) at −80°C within 5 days after collection. Soils were analyzed for soil pH, total carbon, nitrogen phosphorous, and major cations (K, Na, Mg, Ca) at Bemlab, South Africa, using standard procedures.

### DNA extraction and sequencing

Metagenomic DNA was extracted from the soil samples using the DNeasy Powersoil Kit (Qiagen, Valencia, CA, USA) as per the manufacturer’s instructions. DNA samples were submitted for sequencing at a commercial supplier for both metagenome and 16SrRNA sequencing (MR DNA Lab, Shallowater, TX, USA, http://www.mrdnalab.com). Shotgun metagenomic sequencing was performed on a HiSeq 2500 Ultra-High-Throughput Sequencing system (Illumina Inc., San Diego, CA, USA) using paired-ends (2 × 250 bp) for 500 cycles as per the manufacturer’s instructions.

Targeted sequencing of the 16S rRNA gene amplicons were amplified using primers 515F (5′-GTGYCAGCMGCCGCGGTAA-3′) and 806R (5′-GGACTACNVGGGTWTCTAAT-3′). Paired-end 2 × 250 bp sequencing was performed on an Illumina MiSeq instrument according to manufacturer’s instructions (Illumina Inc., San Diego, CA, USA) with the parameters as described (https://support.illumina.com/16s-metagenomic-library-prep-guide-15044223-b.pdf). The metagenome sequence data and 16S amplicon sequence data are available on NCBI (PRJNA592367).

### Metagenome assembly and ARG annotation

Raw reads were quality filtered using FastQC (Andrews, 2010) and trimmed using PrinSeq (Schmieder and Edwards, 2012). Quality reads were assembled using SPAdes v3.12 (Bankevich et al., 2012), with default settings and the “meta” parameter specified. The quality of each assembled metagenome (*n* = 18 ) was assessed using QUAST v5.0.2 (Mikheenko et al., 2018). Gene prediction was performed using Prodigal v2.6.3 (Hyatt et al., 2010) with the “meta” parameter specified. To identify antibiotic resistance genes that may have been acquired by HGT, predicted genes were compared against the ResFinder database (Zankari et al., 2012) by means of BLASTn with an *E*-value threshold of 1 × 10^−6^. The filtering parameters used were 100% similarity and a minimum query length of >50 %. Genes predicted by prodigal were also compared to the comprehensive antibiotic resistance database **(**CARD) (McArthur et al., 2013) by means of BLASTp with an *E*-value threshold of 1 × 10^−6^. Results were filtered for hits with a minimum percentage similarity of 87% and a minimum query length of >40%. These parameters were set with BLAST against all other databases used subsequently.

### Mobile genetic elements and metal/biocide resistance gene annotation

To identify mobile genetic elements flanking ARGS, contigs were compared to the Mobile Genetic Elements Database (Pärnänen et al., 2018) by means of BLASTn. Detected plasmids were confirmed with the PlasmidFinder database (Carattoli et al., 2014). Metal and biocide resistance genes were detected by running BLASTp with these contigs against the BacMet database (Pal et al., 2014). The BacMet database is a manually curated database of antibacterial biocide and metal resistance genes.

### 16S rRNA amplicon sequence analysis

Sequence reads were demultiplexed using Sabre (https://github.com/najoshi/sabre), and primers were removed with cutadapt 2.10 (Martin, 2011). Amplicon sequence variants (ASVs) were resolved using DADA2 version 1.14 (Callahan et al., 2016) in R version 3.6.2 (R Core Team, 2013). Quality filtering and trimming were done using MaxEE = c(2,2) and truncLen = c(220, 200); all other parameters were set to default. The error rates were estimated by learnErrors, and sequences were dereplicated using derepFastq with default parameters. removeBimeraDenovo was used to remove chimeric sequences. Taxonomy was assigned against the SILVA non-redundant database version 138 (https://www.arb-silva.de).

### Data analyses

The analyses were done in R version 3.6.2 using the packages phyloseq (McMurdie and Holmes, 2013), microbiome (Lahti et al., 2019), tidyverse (Wickham et al., 2019), and vegan (Oksanen et al., 2007). ASV alpha-diversity (richness, Shannon, inverse Simpson, Chao1) was calculated using the vegan package in R. Community data matrices were centered log-ratio transformed, and the Euclidian distance measure was used to generate an Aitchison dissimilarity matrix. To reveal the relationship between microbial composition and the resistome, the pairwise Pearson’s rank correlation (correlate alpha-diversity) was calculated, and a Mantel test was conducted directly from the distance matrices (correlate beta-diversity).

## Results and discussion

### Diversity and abundance of ARGs

**Fig. 1:**
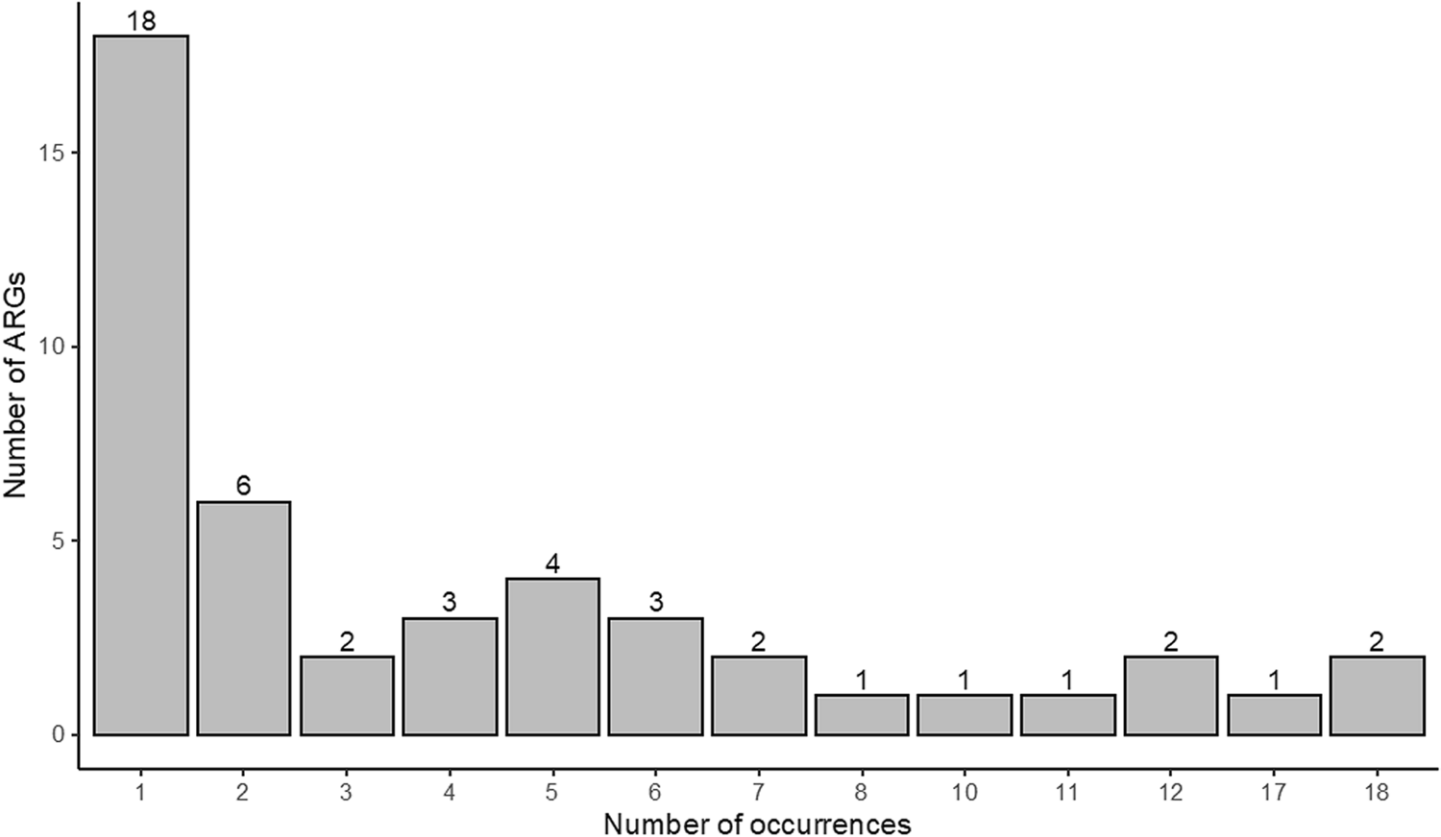
The number of occurrences of unique antibiotic resistance genes (ARGs) at each sample site

We obtained an average of 660,000 ORFs per sample, and the annotation of those ORFs against the CARD database resulted in a total of 6045 ORF hits with the identification of 46 ARGs. The identified ARGs spanned 26 ARG families exhibiting 17 resistance mechanisms (Supplementary Table S1). A great proportion of the ARGs were in low abundance and found in a single sample (Fig. 1), indicating that most ARGs in Namib Desert soils appear to be rare both in terms of local abundance and habitat specificity. Similar results have been found in other deserts (Van Goethem et al., 2018; McCann et al., 2019). At least two factors may contribute to the low diversity and abundance of ARGs in these environments. First, it has been hypothesized that microbial competition in desert soil microbiomes is low; this is because the allocation of most of the available resources is for survival under stressful environmental conditions and/or to the response of moisture availability (Fierer et al., 2012). Second, compared with other environments, deserts generally have very low human (and animal) population density and therefore are less impacted by human anthropogenic activity. Anthropogenic activity has been shown to increase the diversity and abundance of elements of the soil resistome (Wang et al., 2016).

The most common resistance mechanism detected was *target alteration by mutation*, followed by target protection, inactivation mechanisms, and various efflux mechanisms (Supplementary figure S1). Target alteration by mutation typically arises via chromosomal mutation and is therefore not laterally transferred. Mutations in the target-encoding genes often confer multidrug-resistance (D’Costa et al., 2006) and resistance to antibiotics such as cephalosporins (Demaneche et al., 2008) and fluoroquinolones (Riesenfeld et al., 2004). This resistance mechanism is essential for the continued evolution of ARGs to natural and synthetic antibiotics (Woodford and Ellington, 2007), especially in the case of clinically relevant isolates (Munita and Arias, 2016).

**Fig. 2:**
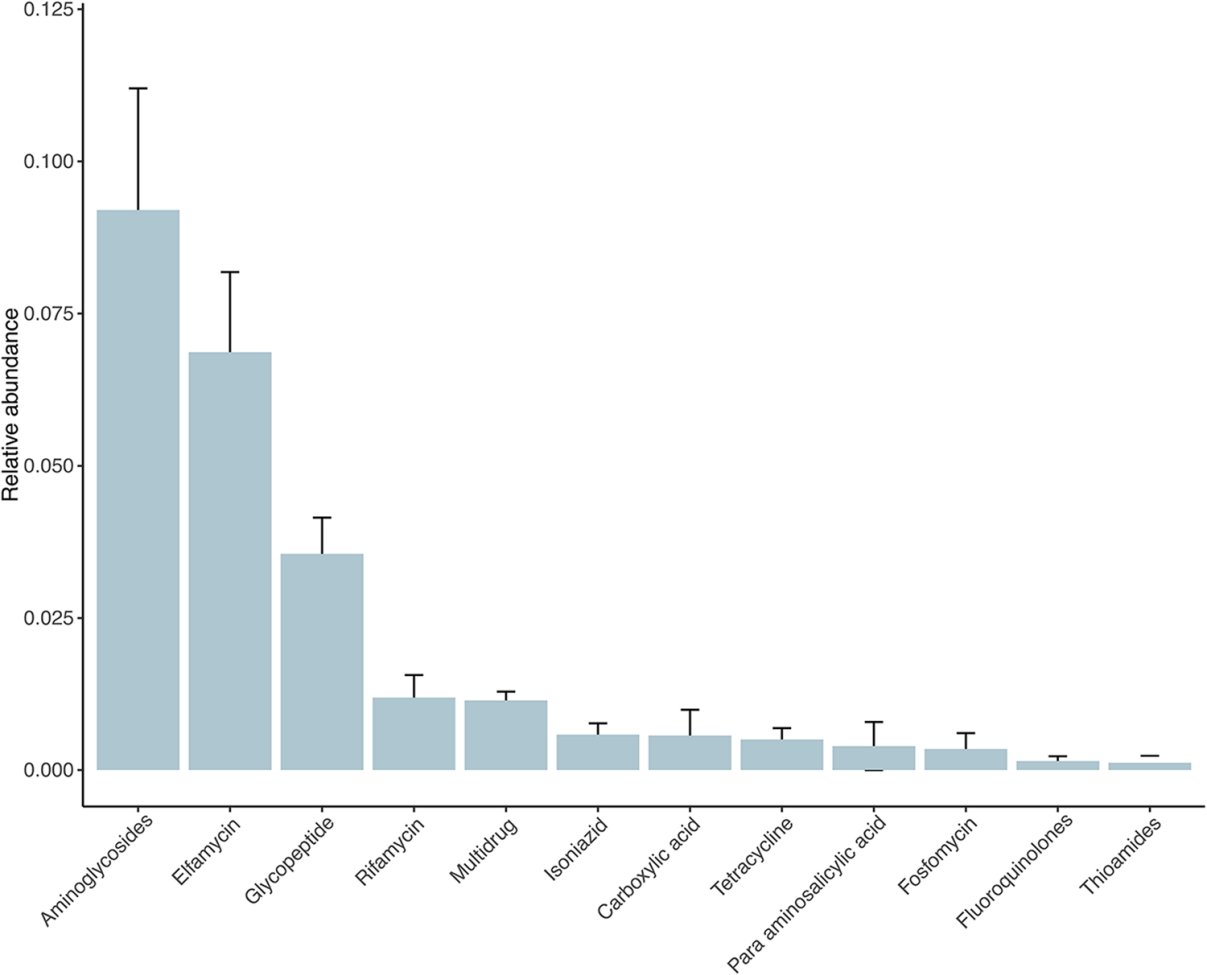
The relative abundance and distribution of ARGs across the Namib Desert soil transect. The error bars represent the standard error of the mean

The most abundant group of ARGs, based on the drug resistance class (Fig. 2), were those that are known to confer resistance to aminoglycosides, elfamycins, glycopeptides, rifamycins, and those that were multi drug resistant. Here, resistance to rifamycins (RIF) was conferred by two different mechanisms (i.e., antibiotic target protection and enzymatic inactivation). Neither of these two mechanisms identified in the Namib soil resistome has been associated with resistance to rifampin in clinical environments (Tomlinson et al., 2016). However, resistance to RIF in these desert soil samples demonstrates that environmental bacteria may also possess multiple mechanisms of resistance to the same antibiotic (Spanogiannopoulos et al., 2012), which is an indication of bacterial adaptation to this environment.

Using the ResFinder database (Zankari et al., 2012), we found two putative horizontally acquired ARGs (Table 1): AAC’3*-la* and *bla*_TEM-116_. AAC’3*-la* (originally detected in clinical strains of *P. aeruginosa*) encodes acetyltransferases that mediate enzymatic inactivation of aminoglycosides. *bla*_TEM-116_ (originally detected in clinical strains of *E. coli* ) is a β-lactamase which showed 100% sequence similarity to the original TEM-116 (Song et al., 2005). Neither bacteriophage sequences nor MGEs were found flanking the AAC’3*-la* genes, and therefore, the vehicle of dissemination could not be established. However, we detected a plasmid (ColE1-like) flanking the *bla*_TEM-116_ gene (Naidoo et al., 2020). This particular variant of TEM-116 was first described on plasmids in clinical isolates in Korea and subsequently on plasmids in several clinical and non-clinical environments (Vignoli et al., 2005; Usha et al., 2008; Maravić et al., 2016).

**Table 1:** Acquired antibiotic resistance genes (ARGs) and the metal/biocide resistance genes (MRGs, BRGs) involved in co-selection of resistance determinants

ARG	Resistance mechanism	Original ARG host	Number of occurrences
AAC(3’)*-la*	Inactivation by acetyltransferase	Clinical strain of *Pseudomonas aeruginosa*	2
*bla*_TEM-116_	Class A β-lactamase, hydrolyzes the peptide bond of β-lactam antibiotics	Clinical strain of *Escherichia coli*	5
MRG and BRG family description	Resistance mechanism	Co-selection compounds	Number of occurrences
*Mex*K	Enhanced Efflux	Triclosan, tetracycline, and macrolides	5
*ars*C	Enzymatic detoxification	Arsenic, third-generation cephalosporins	5

### Metal and biocide resistance genes (MRGs and BRGs) and co-selection with ARGs

**Fig. 3:**
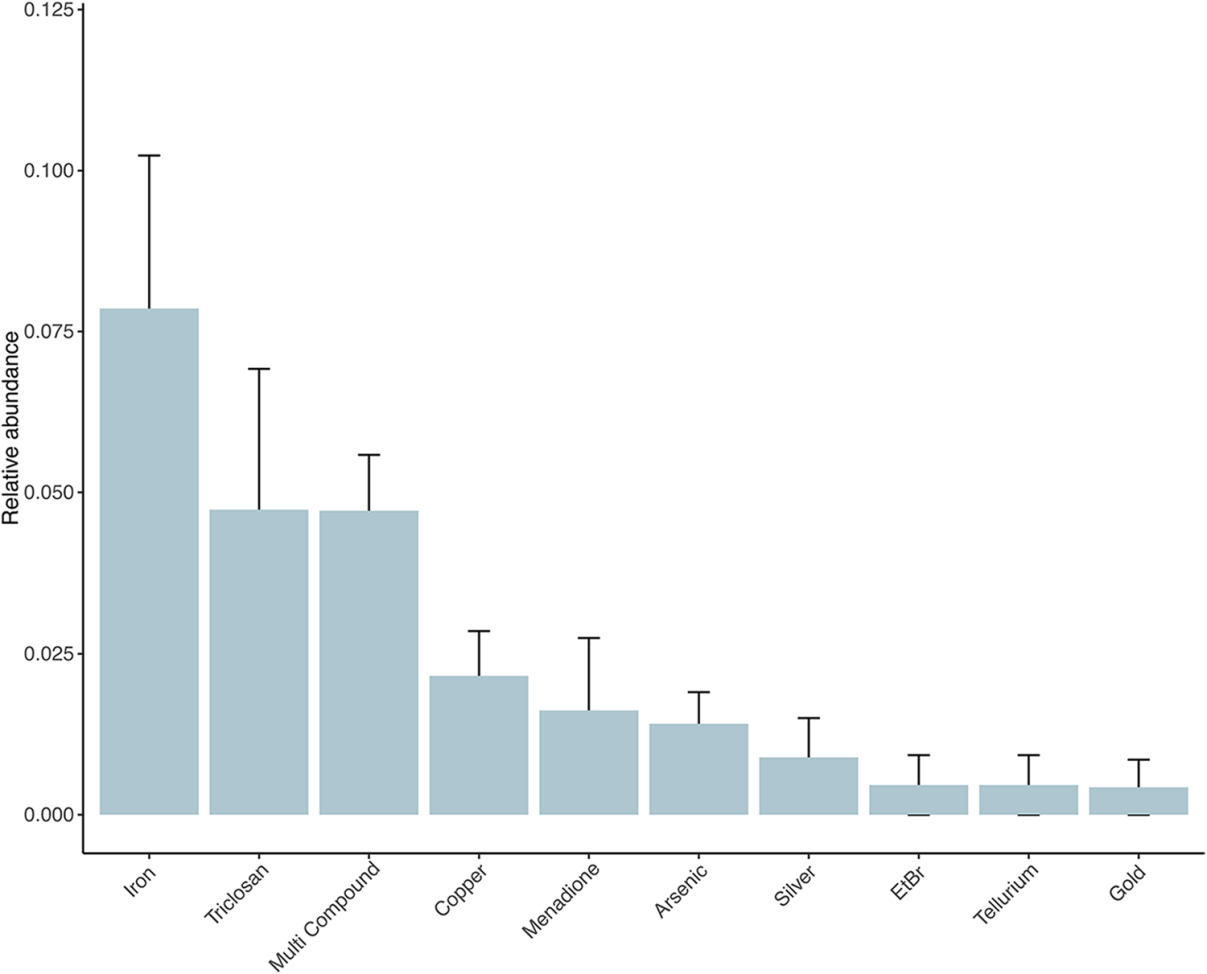
The mean relative abundance and distribution of metal and biocide resistance genes (MRGs and BRGs) across Namib Desert soil transect

The annotation of prodigal-predicted ORFs against the BacMet database resulted in a total of 143 ORF hits with the identification of 29 MRGs and BRGs, which spanned 16 families with 4 known resistance mechanisms (Supplementary Table S2). The most abundant MRGs and BRGs were those that are known to confer resistance to iron and triclosan (biocide) respectively (Fig. 3). Other common identified MRGs were those known to confer resistance to three or more compounds (multi-compound resistance); for example, the resistance gene *Mex*K, which is part of a two-component resistance nodulation cell division (RND) efflux system known as MexJK. This system is responsible for the efflux of triclosan (a phenolic compound used in many personal care products as a biocide) and antibiotics such as macrolides and tetracycline (Chuanchuen et al., 2002). Whether the presence of *Mex*K is indicative of anthropogenic impact in this environment needs to be further investigated.

While antibiotics are readily degraded in soil by multiple mechanisms, metals are essentially non-degradable and therefore potentially impose a long-term selection pressure on microbial communities. In fact, metals and biocides might even exert stronger selection pressure for antibiotic resistance than antibiotics themselves (Pal et al., 2017). The two major mechanisms involved in co-selection of resistance are co-resistance and cross-resistance (Murray et al., 2019). Co-resistance is the genetic linkage of resistance genes; that is, that genes responsible for resistance to two or more compounds are co-located on the same genetic element (i.e., plasmid, transposon or integron) (Seiler and Berendonk, 2012; Pal et al., 2017). An example of co-resistance in this study was the presence of an *ars*C gene on a plasmid that also contained the clinically relevant TEM-116 β-lactamase, suggesting possible co-selection of resistance to antibiotics and arsenic in *Rhodococcus ruber* (Actinobacteria) (Yashini Naidoo et al., 2020). On the other hand, cross-resistance occurs when a resistance gene or a single resistance mechanism simultaneously encodes for resistance to different compounds (Pal et al., 2017; Imran et al., 2019). In these soils, cross-resistance could occur via the MexJK system, which is able to efflux both triclosan and antibiotics such as tetracycline and macrolides (Chuanchuen et al., 2002). The levels of metals in these samples were not measured, but metal concentrations in nearby surface soils (<100 m distance) seem to be high ((i.e., Fe (17 290 ppm), Ni (29.35 ppm), Cu (25.36 ppm), Zn (59.63 ppm), As (3.02 ppm), Ag (0.20 ppm) and U (2.99 ppm)) (Conti et al., 2018). Thus, it is possible that the metals present in this environment may add a selection pressure that could increase the antibiotic tolerance level of microbes via co-selection mechanisms (Imran et al., 2019).

### The occurrence of mobilome-related antibiotic resistance determinants in desert soils

Nine different mobile genetic elements (MGEs) were identified using the Mobile Genetics Elements database. In all metagenomes, insertion sequences were the most abundant MGEs (62% of hits), followed by transposases (33%), integrons (2.7%), and plasmids (2.6%) (Supplementary table S3). The only MGEs linked to any of the ARGs were plasmids (i.e., ColE1 with *bla*TEM-116). The insertion sequences detected were from the families *IS91* and *IS10*. Of the two IS families, the relative abundance of *IS91* sequences was much higher (92%), which might explain the scarcity of MGEs flanking ARGs. Members of the *IS91* family of bacterial insertion sequences are primarily associated with pathogenicity determinants in animals (Schleinitz et al., 2010) and have been reported to very rarely flank ARGs, as they are not suited for the rapid dissemination mechanisms that follow antibiotic exposure in clinical environments (Garcillan-Barcia and De la Cruz, 2002). Conversely, members of the *IS10* family are more rapidly disseminated and are known to upregulate efflux mechanisms resulting in increased antibiotic resistance (Siguier et al., 2014). Here, we identified class 1 integrons (*int1*), which have been a major driver in the spread of antibiotic resistance, specifically in clinical environments (Gillings, 2014). Class 1 integrons have been proposed as markers of anthropogenic pollution due to their common association with resistance to antibiotics and metals in both pathogenic and non-pathogenic bacteria (Gillings, 2014). The presence of *int1* in these soils may infer a moderate human impact

Consistent with previous reports (Wang et al., 2016; Saenz et al., 2019), the overall abundance of MGEs was low. This suggests that only a small fraction of resistance determinants may be mobilized. The horizontal acquisition of resistance genes is suggested to be rare in environments with both low anthropogenic influence and low nutrient levels (Forsberg et al., 2014) as in the case of the Namib Desert. Furthermore, the regulation of genetic machinery is highly responsive to the environment. For instance, the rates of horizontal gene transfer in soils largely depends on variables such as soil moisture and temperature, pH, and soil type (Aminov, 2011). Desert environments experience extreme fluctuations in temperature and highly erratic precipitation patterns, have low nutrient levels, and are generally subject to low levels of anthropogenic impact, compared to other edaphic environments (Makhalanyane et al., 2015). We suggest that these factors together contribute to the observed low abundance of MGEs in Namib Desert soil metagenomes. Nevertheless, the presence of horizontally acquired resistance genes indicates that mobilization of ARGs is a factor in these soils. Like all deserts, the Namib also is impacted by transient wildlife (Stein et al., 2008) that may contribute to the spread of resistance determinants (Allen et al., 2010). It is also noted that over the past few decades, there has been a substantial growth of tourism in the Namib Desert (Woyo and Amadhila, 2018), which may increase the possible routes of transmission for acquired ARGs.

### Decoupling between the microbiome and the resistome

Several studies have shown that bacterial community composition is a key driver that shapes the distribution and abundance of ARGs, for instance, in surface soils (Forsberg et al., 2014), underground coal mine soils (Dunivin and Shade, 2018), and in sewage sludge (Su et al., 2015). However, results from the Mantel test revealed that there were no significant correlations (Mantel *r* = 0.2, *P* > 0.05) between the resistome and the microbiome (ASVs derived from 16S rRNA gene sequences). This suggests that microbial community composition was not a major contributor to the variance in resistance genes in Namib Desert soils and suggest that variations in resistance genes abundance in this soil might be driven by other factors, such as the mobilome. Mobile genetic elements are known to play a very important role in shaping ARG dynamics, as they can transfer genes between distantly related taxa, thereby causing a decoupling of bacterial community and ARG profiles (Fang et al., 2019). Alternatively, a decoupling between the microbiome and the resistome can also be expected if most of the mobile genetic elements are hosted by a limited number of taxa widely distributed across the soil. Further research would be necessary to confirm this.

## Conclusion

This study represents, to the best of our knowledge, the most comprehensive analyses of soil resistomes conducted to date in hot deserts. We have demonstrated that Namib Desert soils contain diverse ARGs and MRGs/BRGs, some of which seem to be acquired on mobile elements. The presence of these acquired resistance genes supports the assumption that vectors such as terrestrial animals, birds, and humans may be partly responsible for the spread of resistance determinants in these soils which highlights the importance of the One Health approach to the burden of antibiotic resistance. In addition, the co-selection of resistance to antibiotics with metals and/or biocides could lead to the persistence of ARGs in the soil microbiome. The presence of ARGs in this soil ecosystem does not necessarily pose a significant health threat to humans and animals living in or visiting this environment. However, the concern is that, like in other environmental settings, the mobilization of these resistance determinants and their expression in bacterial pathogens could present difficulty for treatment options in the clinic.

### Supplementary information

ESM 1 (DOCX 23 kb)

ESM 2 (DOCX 28 kb)

## Data Availability

The metagenome sequence data and 16S amplicon sequence data are available on NCBI (PRJNA592367) and can be accessed at https://www.ncbi.nlm.nih.gov/search/all/?term=PRJNA592367.
