# Supplementary material for: Characterization of the soil resistome and mobilome in Namib Desert soils
Source: Int Microbiol. 2023 Nov 16;27(4):967–75. doi: 10.1007/s10123-023-00454-x (PMC11300574; doi:10.1007/s10123-023-00454-x)
Supplement: Supplementary file 1 — (DOCX 23 kb) [file 10123_2023_454_MOESM1_ESM.docx]

Supplementary tables

Table S1. The number of each ARG detected in the study according to the antibiotic resistance gene family and the corresponding resistance mechanism.

| ARG family Description | Resistance mechanism | Number of ARGs |
| --- | --- | --- |
| rifampin phosphotransferase | antibiotic inactivation by phosphorylation | 1 |
| antibiotic resistant isoleucyl-tRNA synthetase (ileS) | antibiotic target alteration or replacement | 1 |
| rifamycin-resistant beta-subunit of RNA polymerase (rpoB) | antibiotic target alteration or replacement | 3 |
| aminosalicylate resistant dihydrofolate synthase | antibiotic target alteration by mutation | 1 |
| antibiotic resistant inhA | antibiotic target alteration by mutation | 1 |
| antibiotic resistant kasA | antibiotic target alteration by mutation | 1 |
| antibiotic resistant ndh | antibiotic target alteration by mutation | 1 |
| antibiotic resistant rpsL | antibiotic target alteration by mutation | 1 |
| daptomycin resistant beta prime subunit of RNA polymerase (rpoC) | antibiotic target alteration by mutation | 1 |
| elfamycin resistant EF-Tu | antibiotic target alteration by mutation | 4 |
| elfamycin resistant EF-Tu kirromycin self-resistant EF-Tu | antibiotic target alteration by mutation | 1 |
| ethionamide resistant ethA | antibiotic target alteration by mutation | 1 |
| flouroquinolone resistant gyrA | antibiotic target alteration by mutation | 7 |
| fluoroquinolone resistanct gyrB | antibiotic target alteration by mutation | 3 |
| fluoroquinolone resistant parE | antibiotic target alteration by mutation | 1 |
| isoniazid resistant katG | antibiotic target alteration by mutation | 1 |
| murA transferase | antibiotic target alteration by mutation | 1 |
| RbpA bacterial RNA polymerase-binding protein | antibiotic target protection | 1 |
| rifamycin-resistant beta-subunit of RNA polymerase (rpoB) | antibiotic target replacement gene duplication | 1 |
| TEM β-lactamase | class A β-Lactamase | 1 |
| RND antibiotic efflux pump | efflux pump complex | 1 |
| AAC(3’) | inactivation by acetyltransferase | 1 |
| AAC(6’) | inactivation by acetyltransferase | 1 |
| rifampin phosphotransferase | inactivation of rifampin | 1 |
| RND antibiotic efflux pump | metal and multidrug efflux | 1 |
| MFS antibiotic efflux pump | MFS efflux | 2 |
| RND antibiotic efflux pump | multidrug efflux | 1 |
| glycopeptide resistance gene cluster vanR | restructuring of bacterial cell wall | 1 |
| van ligase glycopeptide resistance gene cluster | restructuring of bacterial cell wall | 1 |
| elfamycin resistant EF-Tu | Ribosomal alteration | 1 |
| tetracycline-resistant ribosomal protection protein | ribosomal protection proteins | 1 |
| RND antibiotic efflux pump | RND efflux transporter |  |

Table S2. The number of each MRG detected in the study according to the metal/biocide resistance gene family and the corresponding resistance mechanism.

| Metal/biocide resistance gene family | Resistance mechanism | Number of MRGs |
| --- | --- | --- |
| ABC superfamily | Enhanced Efflux | 1 |
| *ars*B family | Enhanced Efflux | 1 |
| RND superfamily | Enhanced Efflux | 5 |
| Aconitase family | Enzymatic detoxification | 1 |
| *ars*C family | Enzymatic detoxification | 1 |
| Cation transport ATPase | Enzymatic detoxification | 5 |
| Cation transport ATPase (P-type) family | Enzymatic detoxification | 1 |
| Methyltransferase family | Enzymatic detoxification | 1 |
| Multi-copper oxidase family | Enzymatic detoxification | 3 |
| NADPH-dependent FMN reductases family | Enzymatic detoxification | 1 |
| RuvB family | Enzymatic detoxification | 1 |
| Sodium-solute symporter (SSF) family | Membrane transporters | 1 |
| Contains 1 HTH arsR-type DNA-binding domain | Regulatory transporters | 2 |
| Contains 1 HTH dtxR-type DNA-binding domain | Regulatory transporters | 1 |
| Contains 1 response regulatory domain | Regulatory transporters | 1 |
| RND superfamily | Regulatory transporters | 2 |
| *ars*T family | Not yet determined* | 1 |
|  |  |  |

Table S3. The proportion of mobile genetic elements detected in the study.

| Mobile genetic element | Definition | Proportion (%) |
| --- | --- | --- |
| IS10 | Insertion sequence | 1,3 |
| IS10R | Insertion sequence | 0,7 |
| IS91 | Insertion sequence | 60,3 |
| tNiA | Integrase | 0,7 |
| Int2 | Integron | 0,7 |
| istB | Integron | 1,3 |
| ColRNAi | Plasmid | 2,6 |
| Delta-tnpA | Transposase | 0,7 |
| tnpA | transposase | 31,8 |
|  |  |  |
