# Supplementary material for: Characterization of the soil resistome and mobilome in Namib Desert soils
Source: Int Microbiol. 2023 Nov 16;27(4):967–75. doi: 10.1007/s10123-023-00454-x (PMC11300574; doi:10.1007/s10123-023-00454-x)
Supplement: Supplementary file 2 — (DOCX 28 kb) [file 10123_2023_454_MOESM2_ESM.docx]

**Characterization of the soil resistome and mobilome in Namib Desert soils**

Yashini Naidoo^1*^, Rian E Pierneef^2^, Don A Cowan^1^ Angel Valverde^3*^

^1^ Centre for Microbial Ecology and Genomics, Department of Biochemistry, Genetics and Microbiology, University of Pretoria, Lynnwood Road, Pretoria 0002, South Africa

^2^Biotechnology Platform, Agricultural Research Council, Soutpan Road, Onderstepoort Campus, Pretoria 0110, South Africa

^3^IRNASA-CSIC, Cordel de Merinas, Salamanca 37008, Spain

**Supplementary figures:**

**Figure S1.** Prevalence plot for ARG resistance mechanisms. Each point corresponds to a different ARG, the y-axis measures the number of occurrences while the x-axis measures the number of reads.
